# Congruence Amidst Discordance between Sequence and Protein-Content Based Phylogenies of Fungi

**DOI:** 10.3390/jof6030134

**Published:** 2020-08-13

**Authors:** Guohua Xiao, Guirong Tang, Chengshu Wang

**Affiliations:** 1 School of Computer Science, Fudan University, Shanghai 200433, China; guohua.xiao@gmail.com; 2 CAS Key Laboratory of Insect Developmental and Evolutionary Biology, CAS Center for Excellence in Molecular Plant Sciences, Shanghai Institute of Plant Physiology and Ecology, Chinese Academy of Sciences, Shanghai 200032, China; grtang@sippe.ac.cn; 3 CAS Center for Excellence in Biotic interactions, University of Chinese Academy of Sciences, Beijing 100049, China; 4 School of Life Science and Technology, ShanghaiTech University, Shanghai 201210, China

**Keywords:** phylogenomics, protein content phylogeny, cophylogenetic analysis, taxonomic association, lifestyle association

## Abstract

Amid the genomic data explosion, phylogenomic analysis has resolved the tree of life of different organisms, including fungi. Genome-wide clustering has also been conducted based on gene content data that can lighten the issue of the unequal evolutionary rate of genes. In this study, using different fungal species as models, we performed phylogenomic and protein-content (PC)-based clustering analysis. The obtained sequence tree reflects the phylogenetic trajectory of examined fungal species. However, 15 PC-based trees constructed from the Pfam matrices of the whole genomes, four protein families, and ten subcellular locations largely failed to resolve the speciation relationship of cross-phylum fungal species. However, lifestyle and taxonomic associations were more or less evident between closely related fungal species from PC-based trees. Pairwise congruence tests indicated that a varied level of congruent or discordant relationships were observed between sequence- and PC-based trees, and among PC-based trees. It was intriguing to find that a few protein family and subcellular PC-based trees were more topologically similar to the phylogenomic tree than was the whole genome PC-based phylogeny. In particular, a most significant level of congruence was observed between sequence- and cell wall PC-based trees. Cophylogenetic analysis conducted in this study may benefit the prediction of the magnitude of evolutionary conservation, interactive associations, or networking between different family or subcellular proteins.

## 1. Introduction

Phylogenetic analysis, based on DNA or protein sequence data, is critical in evolutionary biology. Amid the genomic data explosion, phylogenomic analysis using a large dataset including hundreds to thousands of orthologous genes/proteins from each species resolved the speciation relationships of different organisms [1]. Molecular phylogenies have also revealed either convergent or divergent evolution of protein family sizes or lifestyles [2]. For example, comparative and phylogenomic analysis of plant, mammalian, and insect pathogenic fungi revealed the convergent evolution of different pathotypes in association with protein family expansions and contractions [3]. Cophylogenetic analysis has also been reported to reveal the co-speciation events between parasites and hosts [4,5]. Thus, tree comparison is beneficial for testing the association or relationships between different phylogenies.

Besides phylogenomic analysis, genome-wide trees also have been generated on the base of the statistic properties of the genome, chromosomal gene order, or genome-wide gene contents [6,7]. For example, clustering analyses using the shared gene contents between bacterial genomes resulted in the phylogenies in good correlation with the sequence-based phylogenetic relationships [8,9,10]. Alternatively, protein contents were converted into binary data for phylogenetic analysis of animals [7,11,12] or viruses [13] based on the presence or absence of homologous gene families after orthology analyses, which also resulted in congruence relationships between sequence- and gene-content-based tress. Phylogenetic clustering using these data could alleviate the issue of the unequal evolutionary rate of the genes used for phylogenomic analysis [14] or the issue of the substantial gene turnover between deeply diverging lineages [7]. However, the reliability of the gene content tree has been questioned for resolving speciation relationships due to the events of horizontal gene transfer [6] or gene duplications (i.e., the presence of multiple paralogs for certain genes) [7]. It is still obscure for distance-based phylogenetic analysis once the whole genome gene content was divided into protein families or subcellular locations. The congruent or discordant relationship between sequence and protein family-content based phylogenies has yet to be determined.

The analysis of the gene/protein content tree has not reported before for fungi. By using cross-phylum fungal species as models, the aim of this study is to compare the sequence-based phylogenomic tree with the protein content (PC)-based trees generated based on the PC matrices of the protein families and subcellular location classifications. The results of cophylogenetic analysis may benefit the understanding of the congruent or discordant relationships among these two types of trees and therefore the level of associations or disassociations between protein families.

## 2. Material and Methods

### 2.1. Examined Fungal Species

Whole genome protein sequences of 27 fungal species belonging to three phyla, i.e., Mucoromycota, Basidiomycota and Ascomycota, were downloaded from available resources (Table S1). These species were selected and included in this study based on the considerations of: availability of genome information, different pathotypes, and taxonomic associations. In addition to the model saprophytic fungal species such as *Neurospora crassa* (abbreviated: NCR), *Aspergillus nidulans* (AND), *Saccharomyces cerevisiae* (SCE), and *Schizosaccharomyces pombe* (SPO), cross-phylum plant pathogens like the ascomycetes *Magnaporthe oryzae* (MOR), *Fusarium graminearum* (FGR), *Sclerotinia sclerotiorum* (SSC), *Verticillium albo-atrum* (VAL), and *Botrytis cinerea* (BCI), as well as the basidiomycete *Ustilago maydis* (UMA), were selected. Likewise, the cross-phylum mammalian pathogens, including the ascomycetes *A. fumigatus* (AFU), *Coccidioides immitis* (CIM), and *Candida albicans* (CAL), and the basidiomycete *Cryptococcus neoformans* (CNE), were selected. Insect pathogens included two closely-related species with different host range: *Metarhizium robertsii* (MAA) and *M. acridum* (MAC) [5]. Taken together with the selection of four species of the basidiomycete mushrooms, the use of these fungal groups may benefit the determination of lifestyle association. Otherwise, four *Aspergillus* species and three ascomycete yeast species were selected based on the consideration of their taxonomic associations. The basal mucoromycete saprophyte *Rhizopus oryzae* (ROR) was included to root the phylogenetic trees.

### 2.2. Pfam Analysis and Subcellular Location Prediction

Proteins encoded by each fungal species were first subject to Pfam analysis and classified based on the top hit with a cutoff of *E* value < 1 × 10^−5^ (Table S2). A few large protein families were then extracted including proteases, CAZy (carbohydrate-active enzymes) [15], ribosomal subunits, and those proteins with domain-unknown function (DUF) and uncharacterized protein family (UPF) (Tables S3–S6). Subcellular localizations of the proteins of each species were analyzed using the program ProtComp (ver. 9.0; http://linux1.softberry.com/berry.phtml/). Each protein location was determined based on the highest hit score obtained among different categories. Fungal cell wall proteins of each species were also characterized using the algorithm ProFASTA (La Mancha, Spain) [16]. The obtained proteins for each subcellular location were then subject to Pfam annotation. The identified proteins without conserved Pfam domains were not included in further analysis. Manual curations were conducted to remove those Pfam classes present only in one or two fungal species that might be caused due to the gene annotation errors. Thus, protein family sizes were obtained for each subcellular proteins of each fungal species (Tables S7–S16).

### 2.3. Phylogenetic Tree Construction

To rebuild the phylogenomic tree, 455 single-copy orthologous proteins were identified from each of 27 examined fungal species by Inparanoid analysis [17] (Table S17). Protein sequences were aligned using the program MUSCLE (ver. 3.8.31; Mill Valley, USA) [18], and the maximum likelihood (ML) tree was generated using the concatenated sequences with the program TreePuzzle (ver. 5.2; Berlin, Germany) with default parameters including a Dayhoff model of substitution and 1000 bootstrap replicates [19]. Fungal phenograms were also constructed by hierarchical clustering [8] using the average distance matrices estimated from pairwise Pearson correlation coefficient calculated based on the protein content (PC) of different families (Tables S2–S16). The analysis was conducted in a MATLAB TAH Edition (https://github.com/knowledgeontology/fungal_evolution).

### 2.4. Tree Topology Congruence Tests

To determine the significance of topological congruence or incongruence between sequence and PC-based phylogenetic trees, the Newick tree format data (Table S18) were tested by calculation of the index *Icong* based on maximum agreement subtrees without parametrizing the likelihood of evolutionary events. A *p*-value less than 0.05 means that two trees are more congruent than expected by chance [20].

## 3. Results and Discussion

### 3.1. Protein Family Size Variation

Pfam analysis identified 2806 protein families with content variations for each Pfam class from 27 examined fungal species (Table S2), including 49 families of proteases (Table S3), 64 families of CAZy enzymes (Table S4), 94 classes of ribosomal subunits (Table S5), and 344 classes of DUF/UPF proteins (Table S6). Pfam analysis could not finely classify some big protein families such as kinases, transporters, transcription factors, and cytochrome P450s. The contents of these families were therefore not included in further analysis. Subcellular classifications resulted in the varied number of the proteins with putative locations to different organelles for each fungal species (Tables S7–S16). Overall, for example, plant pathogenic and white-rot fungi encode more CAZy enzymes than do other fungi. These glycoside hydrolases are essential for these fungi to degrade plant biomasses [21]. In contrast, higher numbers of chitinases (PF00704, Glycos_hydro_18) and serine proteases (PF00082, Peptidase_S8; PF00089, Trypsin) are encoded in insect pathogenic *Metarhizium* species than those of other fungi. These enzymes are important for *Metarhizium* to degrade and penetrate the chitin- and protein-rich insect cuticles [22]. Overall, similar to previous observations [23,24], protein family content largely correlates with fungal lifestyles.

### 3.2. Diverse Phylogenetic Associations

**Figure 1:**
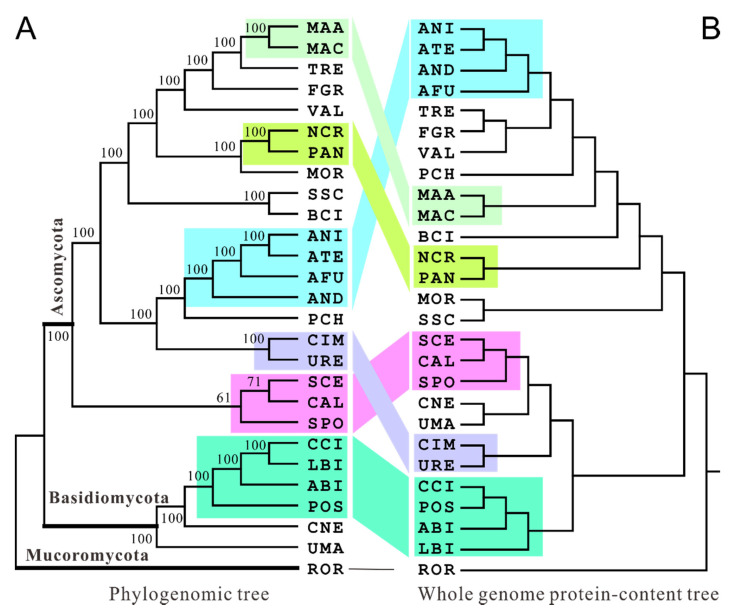
Cophylogenetic analysis of sequence (**A**) and whole genome protein-content-based (**B**) trees. The similarly grouped lineages in both types of trees are highlighted in the same color and connected. The abbreviations of fungal species are listed in Table S1 if not mentioned in the context.

A ML phylogenomic tree was generated (Figure 1A) using the concatenated orthologous protein sequences. Being supported by bootstrap replications and consistent with previous reports [25,26], the tree is rooted by *R. oryzae*, which is then followed by the sequential divergence of basidiomycete and ascomycete fungi. Within the ascomycete lineage, yeast fungi diverged first while insect pathogenic *Metarhizium* species evolved after the speciation of plant pathogens (Figure 1A). Consistent with a previous report [27], the penicillin-producing fungus *Penicillium chrysogenum* (PCH) is grouped with *Aspergillus* species with *A. nidulans* being diverged first. Thus, a reliable phylogenetic tree was obtained.

Based on the selected protein family and subcellular PC matrices, 15 phenogram trees were also generated with different topologies (Figures S1 and S2; Table S18). Of these, only the trees generated for the whole genome, nuclear, mitochondrial, and plasma membrane protein contents could be rooted to *R. oryzae* (Figure 1B and Figure S2). It was also found that, in contrast to the phylogenetic tree, sequential divergence of the basidiomycete and ascomycete fungi from the basal species *R. oryzae* could not be perfectly evident in any PC-based phylogeny. However, lineage-specific clustering was frequently observed for those fungi with either the same lifestyles (e.g., plant, insect, or human pathogens) or taxonomic connections (e.g., the ascomycete *Aspergillus* and yeast species). For example, two insect pathogenic *Metarhizium* species (i.e., *M. robertsii* (MAA) and *M. acridum* (MAC) [5], and two closely-related necrotrophic fungal plant pathogens *S. sclerotiorum* (SSC) and *B. cinerea* (BCI) [28] were frequently clustered together in different PC-based trees (Figures S1 and S2). In particular, the basidiomycete human pathogen *C. neoformans* (CNE) could even be grouped with the ascomycete human pathogen *C. immitis* (CIM) and its close relative *Uncinocarpus reesii* (URE) [29] in nuclear and mitochondrial PC-based trees (Figure S2). Overall, similar to the observation in bacteria [30], PC-based phylogenies usually grouped fungi according to fungal lifestyles rather than their evolutionary relationships. However, considering that the closely related species SSC and BCI, CIM and URE, and even the yeast species are not always clustered together in different trees, the results also implicated that the species grouped in the same lineage do not always have more similar PC distributions than for species in different branches.

### 3.3. Congruence and Discordance Relationships of Different Phylogenies

Having obtained the sequence- and PC-based phylogenies, we next conducted the similarity tests between tree topologies by calculation of congruence indices in pairs. As a result, only protease- and DUF/UPF-PC trees were reliably (*p* < 0.01) congruent than expected by chance with the phylogenomic tree; intriguingly, the significance levels were higher than those between the whole genome PC and sequence-based trees (Table 1 and Figure 1B). It was also intriguing to find that the function-unknown PC-based tree was highly congruent (*Icong* = 1.45; *p* = 1.36 × 10^−3^) with the whole genome PC phylogeny, and the CAZy PC tree only tightly correlated (*Icong* = 1.71; *p* = 2.20 × 10^−5^) with the protease PC phylogeny (Table 1). Unexpectedly, it was found that the tree generated from the highly conserved ribosomal subunit content was incongruent to any other phylogenies. Our results suggested therefore that the tree topological associations would vary in a case-dependent manner but reflect to some extent the links between different protein families. For example, the size distribution of the highly cophylogenetic proteases and CAZy enzymes have been shown with essential roles in determining the adaptation of fungi to different niches including hosts [24,31]. It has been found that the genes/proteins with family size conservation evolved more slowly than those with frequent size changes [32]. In this respect, cophylogenetic analysis between sequence- and PC-based trees might benefit the prediction of the gene family evolutionary rates, i.e., the lower the *Icong* index value, the slower the evolutionary speed. The high *Icong*-index values obtained between DUF/UPF and sequence or other protein family PC-based trees suggested that these proteins were fast evolving and would play important roles in fungal physiology with other protein families.

**Figure 2:**
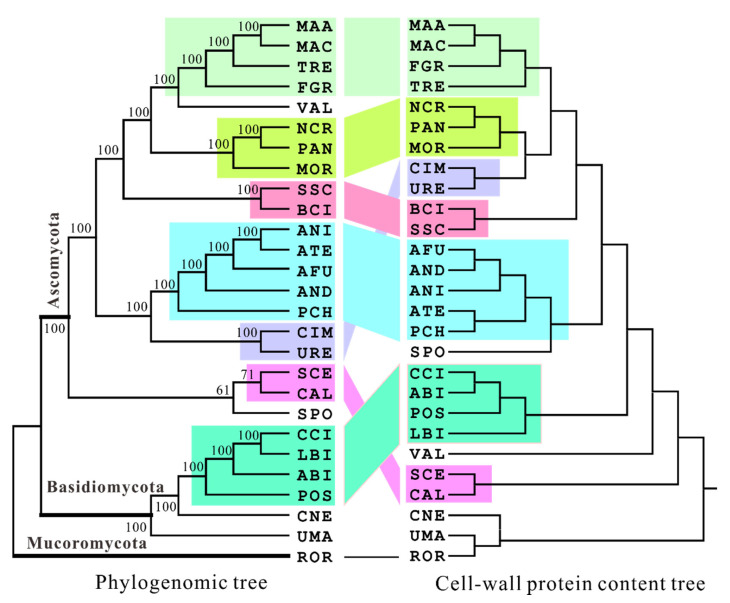
Cophylogenetic analysis of sequence (**left**) and cell wall protein-content-based (**right**) trees. The similar grouped lineages in both types of trees are highlighted in the same color and connected. The abbreviations of fungal species are listed in Table S1 if not mentioned in the context.

We next tested the congruent relationships between sequence and subcellular PC-based trees. It was found that, unlike the relatively low levels of protein family PC-based cophylogenetic associations (Table 1), nine of eleven subcellular PC-based trees were significantly (*p* < 0.05) congruent with the phylogenomic tree except for the vacuolar and lysosomal PC phylogenies (Table 2). In terms of the significance level, the cell wall PC-based phylogeny was mostly similar (*p* = 3.56 × 10^−7^) to the sequence tree (Figure 2), which was even higher than that between phylogenomic and the whole genome PC-based trees (*p* = 0.0106). The cell wall PC tree had seven lineages being similar to those grouped in the phylogenomic tree, whereas the whole genome PC tree had six similarly branched lineages with the sequence tree; in particular, the *Aspergillus* and *Metarhizium* lineages could not be correctly positioned like the phylogenomic tree (Figure 1 and Figure 2). In addition, we found that the whole genome PC tree even has a lower *p* value than those of the nuclear and mitochondrial PC-based trees when compared with the sequence-based phylogeny (Table 2). Consistent with a previous finding that the evolutionary distance between two fungal species significantly correlated with the divergence of their cell wall proteins [33], a high level of congruence between sequence- and cell wall PC-based trees suggested that cell wall proteins might be more highly associated with fungal speciation phylogeny than other organelle proteins.

The tests among subcellular PC-based phylogenies indicated that, except for vacuolar and lysosomal trees, the cophylogenetic relationships were largely congruent among different organelle PC-based trees, among which the most highly congruent (*Icong* = 2.11; *p* = 4.54 × 10^−9^) relationships were observed between the whole genome and cytoplasm trees, and between mitochondrial and Golgi trees (Table 2). Theoretically, all organelles and their associated proteins, even dynamically changing and networking, are functionally important for cell physiology. In particular, fungal cell wall proteins are rapidly changing in different environments to play frontline roles during interaction with different niches including hosts [34]. Both vacuole and lysosome are acidic compartments that can change in shape, number, and size in different fungi [35]. Consistently, the highly variable numbers of proteins belonging to different families were observed for these two organelles (Tables S15 and S16), which might result in the abnormal topology of their PC trees and, therefore, the cophylogenetic discordance with other trees. Otherwise, the varied level of cophylogenetic associations between different subcellular PC datasets may reflect the magnitude of interactive association or networking between different organelle proteins.

## 4. Conclusions

Using different fungal species as models, we report both the congruence and discordance relationships between sequence- and PC-based phylogenies that could more or less reflect the lifestyle and taxonomic associations between closely related fungi. However, unlike previous suggestions [8,36], it is evident in this study that the whole genome and or separate PC-based phylogenetic analysis cannot resolve the phylogenetic trajectory of cross-phylum fungal species. Cophylogenetic analysis of the sequence- and PC-based trees may benefit the prediction of the magnitude of evolutionary conservation, interactive associations, or networking between different subcellular or family of proteins. Further analysis is still required to either convert the PC dataset into binary data for bootstrap analysis [7] or use a likelihood method to accommodate the peculiarity of the discrete PC datasets [37]. It can be expected that the association levels may vary when more fungal species and or additional protein families have been included in analysis, which is worthwhile for future investigations including the incorporation of different kingdom organisms.

## Figures and Tables

**Table 1:** Congruence tests among the sequence and selected protein family size-based trees *.

	Sequence Tree	Genome-Wide Proteins	Proteases	CAZy **	Ribosomal Subunits
Whole genome proteins	1.32; 0.0106				
Proteases	1.45; 1.36 × 10^−3^	1.19; 0.0835			
CAZy **	1.19; 0.0835	1.19; 0.0835	1.71; 2.20 × 10^−5^		
Ribosomal subunits	1.05; 0.656	1.19; 0.0835	1.05; 0.656	1.19; 0.0835	
DUFs and UPFs ***	1.45; 1.36 × 10^−3^	1.71; 2.20 × 10^−5^	1.45; 1.36 × 10^−3^	2.11; 4.54 × 10^−8^	1.19; 0.0835

* The data in each cell represent the *Icong* and *p* values. ** CAZy, carbohydrate active enzymes. ***, DUF, protein with domain unknown function; UPF, uncharacterized protein family.

**Table 2:** Congruence tests among the sequence and subcellular protein-content-based trees *.

	Sequence Tree	WGP	CWP	NP	MP	CP	PMP	ERP	GP	EP	VP **
Whole genome proteins (WGP)	1.32; 0.0106										
Cell wall protein (CWP)	1.98; 3.56 × 10^−7^	1.32; 0.0106									
Nuclear protein (NP)	1.71; 2.20 × 10^−5^	1.58; 1.72 × 10^−4^	1.85; 2.80 × 10^−6^								
Mitochondrial protein (MP)	1.58; 1.72 × 10^−4^	1.85; 2.80 × 10^−6^	1.58; 1.72 × 10^−4^	1.85; 2.80 × 10^−6^							
Cytoplasmic protein (CP)	1.45; 1.36 × 10^−3^	2.11; 4.54 × 10^−8^	**1.06;** **0.6560**	1.32; 0.0106	1.71; 2.20 × 10^−5^						
Plasma membrane protein (PMP)	1.45; 1.36 × 10^−3^	1.71; 2.20 × 10^−5^	1.85; 2.80 × 10^−6^	1.58; 1.72 × 10^−4^	1.98; 3.56 × 10^−7^	1.85; 2.80 × 10^−6^					
Endoplasmic reticulum protein (ERP)	1.32; 0.0106	1.45; 1.36 × 10^−3^	1.32; 0.0106	1.45; 1.36 × 10^−3^	1.98; 3.56 × 10^−7^	1.45; 1.36 × 10^−3^	1.85; 2.80 × 10^−6^				
Golgi protein (GP)	1.32; 0.0106	1.58; 1.73 × 10^−4^	1.58; 1.73 × 10^−4^	1.58; 1.73 × 10^−4^	2.11; 4.54 × 10^−^^8^	1.58; 1.72 × 10^−4^	1.71; 2.20 × 10^−5^	1.71; 2.20 × 10^−5^			
Extracellular protein (EP)	1.32; 0.0106	1.45; 1.36 × 10^−3^	1.58; 1.73 × 10^−4^	1.71; 2.20 × 10^−5^	1.85; 2.80 × 10^−6^	1.71; 2.20 × 10^−5^	1.85; 2.80 × 10^−6^	1.71; 2.20 × 10^−5^	1.58; 1.72 × 10^−4^		
Vacuolar protein (VP)	**1.19;** **0.0835**	**1.19;** **0.0835**	**1.06;** **0.6560**	1.32; 0.0106	1.45; 1.36 × 10^−3^	1.32; 0.0106	1.58; 1.72 × 10^−4^	1.32; 0.0106	1.32; 0.0106	1.45; 1.36 × 10^−3^	
Lysosomal protein (LP)	**0.92;** **5.15**	1.32; 0.0106	1.32; 0.0106	1.32; 0.0106	**1.19;** **0.0835**	1.32; 0.0106	1.32; 0.0106	1.45; 1.36 × 10^−3^	**1.19;** **0.0835**	1.45; 1.36 × 10^−3^	**1.19;** **0.0835**

* Two row data in each cell represent the *Icong* (upper) and *p* (lower) values. The values highlighted in bold indicate the paired trees are not more congruent than expected by chance (i.e., *p* > 0.05). ** abbreviations in this title row are as shown in the far left column.

## Supplementary Materials

The following are available online at https://www.mdpi.com/2309-608X/6/3/134/s1, Figure S1: Phylogenetic construction of the examined fungal species based on protein-family size distribution, Figure S2: Phylogenetic construction of the examined fungal species based on subcellular protein content, Table S1: Information of the examined fungal species, Table S2: Pfam matrix of all proteins from each fungal species, Table S3: Pfam matrix of proteases identified from each fungal species, Table S4: Pfam matrix of CAZy enzymes identified from each fungal species, Table S5: Pfam matrix of ribosomal subunits identified from each fungal species, Table S6: Pfam matrix of DUF and UPF proteins identified from each fungal species, Table S7: Pfam matrix of cell wall proteins identified from each fungal species, Table S8: Pfam matrix of nuclear proteins from each fungal species, Table S9: Pfam matrix of mitochondrial proteins from each fungal species, Table S10: Pfam matrix of cytoplasmic proteins from each fungal species, Table S11: Pfam matrix of plasm membrane proteins from each fungal species, Table S12: Pfam matrix of endoplasmic reticulum proteins from each fungal species, Table S13: Pfam matrix of Golgi proteins from each fungal species; Table S14: Pfam matrix of extracellular proteins from each fungal species, Table S15: Pfam matrix of vacuolar proteins from each fungal species, Table S16: Pfam matrix of lysosomal proteins from each fungal species, Table S17: One-to-one orthologous genes identified from each fungal species (the accessions for each species are retrieved from the respectively curated databases), Table S18: Newick tree datasets generated from the orthologous sequences and different protein-content data.
