# Supplementary material for: Congruence Amidst Discordance between Sequence and Protein-Content Based Phylogenies of Fungi
Source: J Fungi (Basel). 2020 Aug 13;6(3):134. doi: 10.3390/jof6030134 (PMC7559059; doi:10.3390/jof6030134)
Supplement: Supplementary file 1 [file jof-06-00134-s001.zip › SuppFigs.docx]

**Supporting Figures**


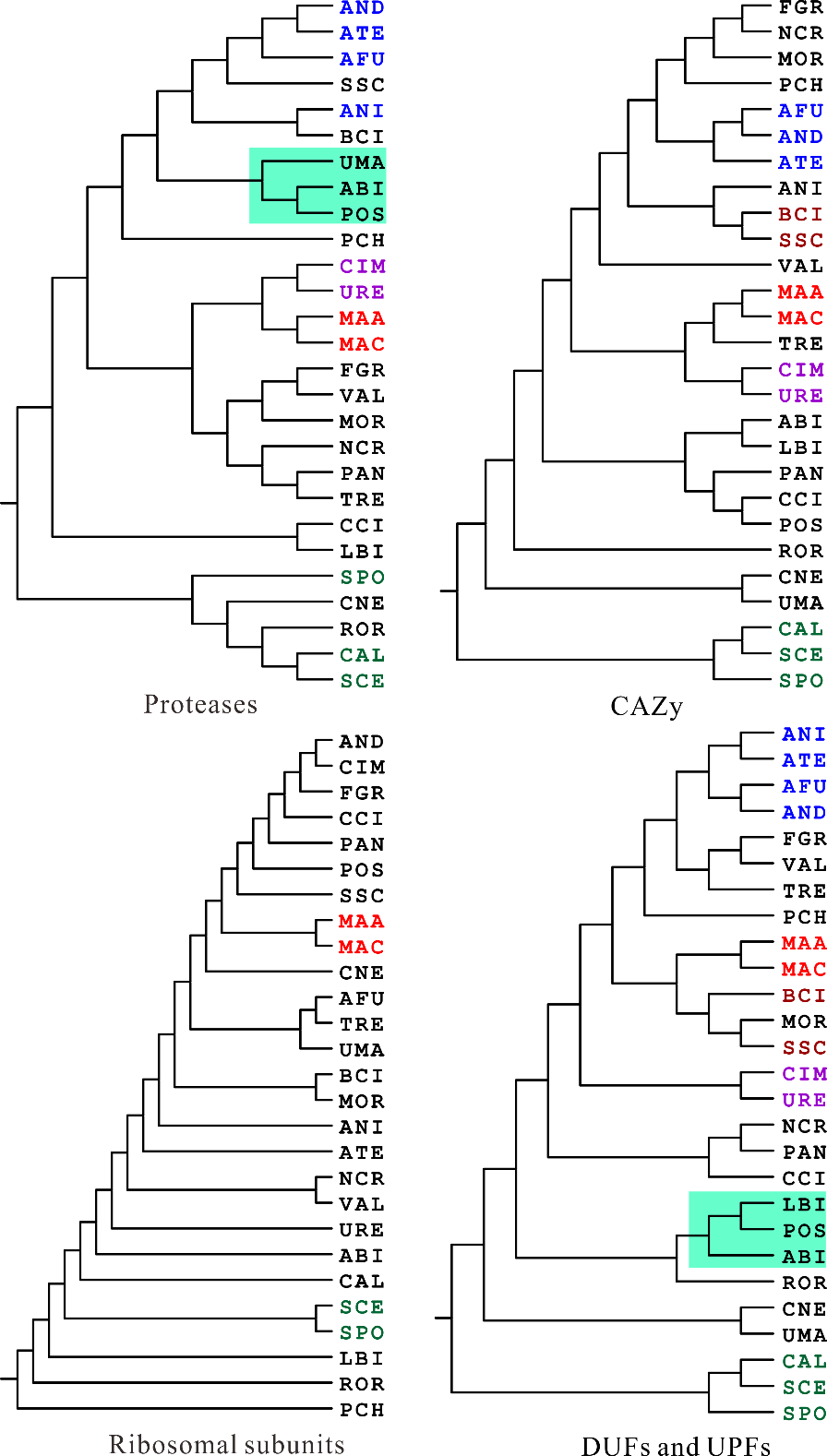


**Figure S1.** Phylogenetic construction of the examined fungal species based on protein-family size distribution. The protein content of each selected protein families was based on Pfam analysis. The abbreviation of each fungal species is listed in Table S1. The species showing similar grouping patterns with the molecular speciation tree are labeled in the same color or shadowed.


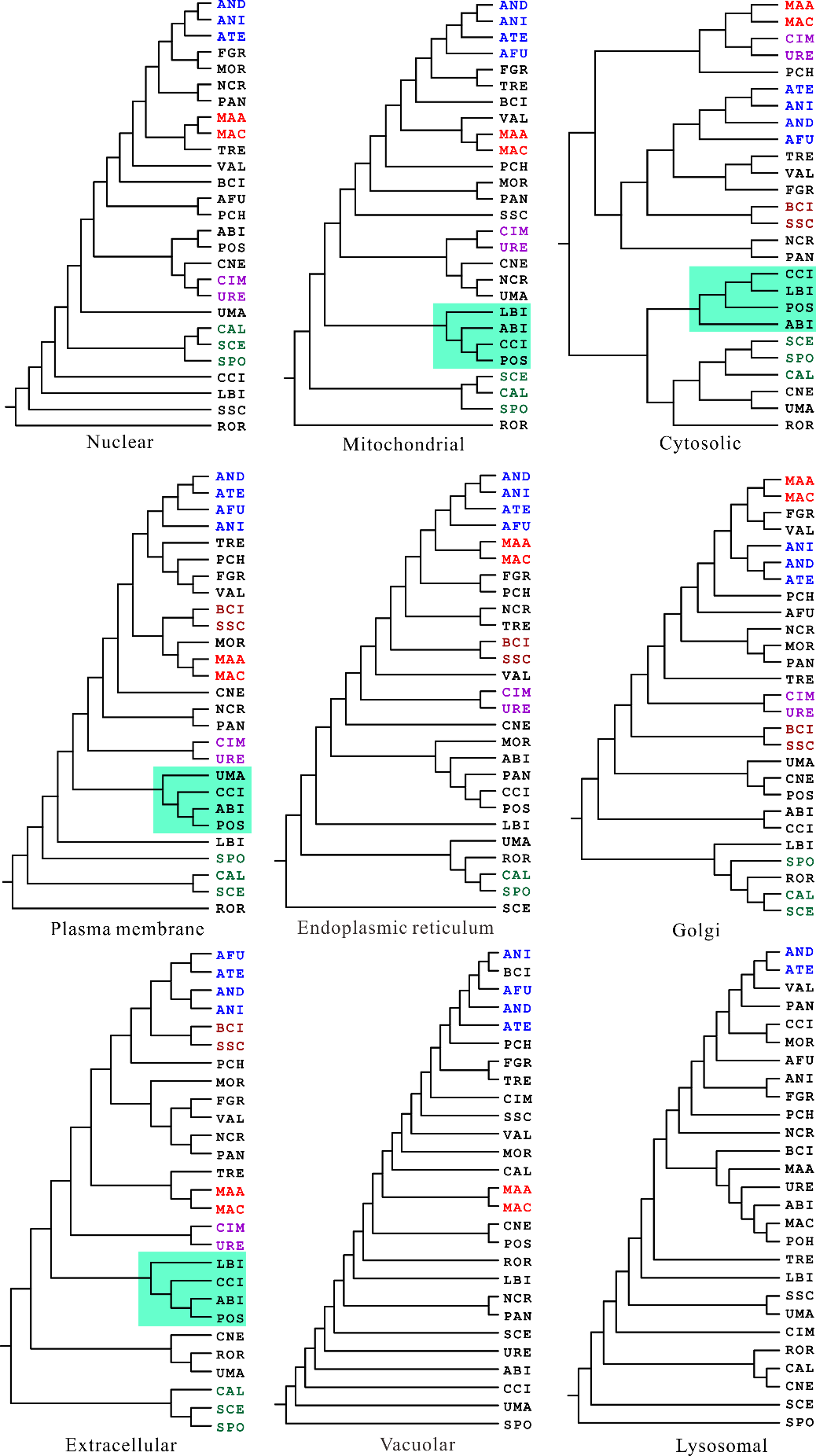


**Figure S2.** Phylogenetic construction of the examined fungal species based on subcellular protein content. The abbreviation of each fungal species is listed in Table S1. The species showing similar grouping patterns with the molecular speciation tree are labeled in the same color or shadowed.
